# Supplementary material for: Determinants and strategies for environmental compliance in municipalities: Perspectives from KwaZulu-Natal Province, South Africa
Source: PLoS One. 2026 Jan 16;21(1):e0338709. doi: 10.1371/journal.pone.0338709 (PMC12810821; doi:10.1371/journal.pone.0338709)
Supplement: S1 File — (PDF) [file pone.0338709.s001.pdf]

Annexure A-Interview guides

Annexure A1-Interview guide for municipal officials

Date, time, and place of the interview

Interviewee details

Name, gender, age, race, qualifications, designation, marital status and years in service

1. Tell me about your current role and responsibilities relating to environmental management?
2. How would you describe your municipality's approach to environmental governance and compliance?
3. How are environmental compliance responsibilities structured with the municipality?
4. How does leadership within the municipality influence environmental compliance decisions?
    a. Probes: Are decisions influenced by political or administrative instructions?
    b. Probes: How do council decisions impact compliance behaviour and resource allocation?
5. What are the main determinants of environmental compliance in municipalities?
6. What factors affect compliance in municipalities?
7. How do municipalities engage with stakeholders in addressing environmental issues?
8. In your view, what are some institutional changes that could improve environmental compliance in municipalities?
9. Is there anything else you would like to add that we have not discussed?

Annexure A2 -interview guide Environmental Management Inspectors and Environmental Consultants

Date, time, and place of the interview

Interviewee details

Name, gender, age, race, qualifications, designation, marital status and years in service

1. Tell me about your current role and responsibilities relating to environmental management?
2. What comes to mind when you hear someone talk about environmental compliance in municipalities.
3. How are environmental compliance responsibilities structured within the municipalities you have worked with?
4. How does leadership within the municipality influence environmental compliance decisions?
    a. Probes: Are decisions influenced by political or administrative instructions?
    b. Probes: How do council decisions impact compliance behaviour and resource allocation?
5. What are the main determinants of environmental compliance in municipalities?
6. What factors affect compliance in municipalities?
7. How do municipalities engage with you and other stakeholders in addressing environmental issues?
8. In your view, what are some institutional changes that could improve environmental compliance in municipalities?
9. Is there anything else you would like to add that we have not discussed?

University of South Africa
Preller Street, Muckleneuk Ridge, City of Tshwane
PO Box 392 UNISA 0003 South Africa
Telephone: +27 12 429 3111 Facsimile: +27 12 429 4150
www.unisa.ac.za
