# Supplementary material for: Determinants and strategies for environmental compliance in municipalities: Perspectives from KwaZulu-Natal Province, South Africa
Source: PLoS One. 2026 Jan 16;21(1):e0338709. doi: 10.1371/journal.pone.0338709 (PMC12810821; doi:10.1371/journal.pone.0338709)
Supplement: Attachment — Submitted filename: Response to Reviewers_PONE-D-25-23589.docx [file pone.0338709.s003.docx]

**Response to Reviewers-PONE-D-25-23589**

| We sincerely thank the Editor and Reviewers for their constructive comments and for recognizing the potential contribution of our manuscript to PLOS ONE. We have carefully considered each point raised and made substantial revisions to enhance the clarity, rigor, and theoretical coherence of the paper. Below, we provide a detailed, point-by-point response indicating the changes made. | |
| --- | --- |
| 1. Title and Abstract the abstract provides a concise summary, but would benefit from clearly separated sections (e.g., Background, Methods, Results, Conclusion) to improve reader accessibility and conform with best practice. I would suggest the authors to reformat the abstract into structured subheadings to enhance clarity. | We have revised the abstract and restructured it to sub-headings; Background, Methods, Results, Conclusion. Each of these sections have also been refined for brevity and focus. |
| 2. Introduction  - However, lines 49–56 should be restructured to clarify the distinction between compliance vs. enforcement and the challenge of “fixed rules” in sustainability. I would suggest that the authors to briefly define environmental compliance versus enforcement and highlight examples where legislation may fall short due to institutional fragmentation. | We have edited the introduction from the beginning to the end to clarify the gap, and improve its coherence with the conclusion.  We have defined environmental compliance and included factors that may make municipalities remain non-compliant regardless of the legislation. |
| 3. Theoretical Framework  - The integration of governance theory (GT) is appropriate and enhances the conceptual depth of the study. - However, the connection between GT and the study’s coding themes could be strengthened in the discussion. The authors should explicitly link themes like political support, enforcement, and institutional restructuring to GT’s dimensions (e.g., hierarchical, networked, market-based governance) in the discussion section. | We have improved the connection between the findings and the GT approaches. We have improved interpretation to specify the type of GT approach used in municipalities, the approach proposed by ECs and EMIs. |
| 4. Methodology  - Ethical protocols, data collection, and participant demographics are clearly presented. - The summary of methods lacks sufficient detail on: - Interview guide development, - Justification for the sample size (n = 27), and - Coding reliability/validation measures. - The authors should include a supplementary file with the semi-structured interview guide. Clarify whether inter-coder agreement or peer debriefing was used to validate themes. | Thank you for this constructive observation.  We have revised the methods section to give more details regarding ethical guidelines followed, we have included the interview guide as Annexure A, justified the sample size and explained measures for coding reliability. We also indicated the application of peer debriefing to maintain interpretive transparency. |
| 5. Results  - Results are rich and supported with meaningful quotes. - The inclusion of Figures 1–3 is commendable, especially: - Figure 1 (page 34): Compliance drivers by role group, - Figure 2 (page 35): Co-occurrence of drivers, and - Figure 3 (page 36): Preferred interventions by stakeholder group. - However, the narrative tends to over-quote participants. Several quotes repeat the same theme. Focus on integrating fewer but more representative quotes and enhance comparative analysis between participant categories (e.g., ECs vs EMIs). | Thank you for this constructive observation. The results section has been revised to reduce the number of direct quotations and to include only those that are most representative and illustrative of each major theme. Repetitive quotes conveying similar sentiments have been removed. |
| 6. Discussion  - The discussion aligns well with findings and relevant literature. - Some claims are too general (e.g., “political will is a cornerstone…”), and citations need to be used more selectively. - The link between findings and institutional theory could be sharpened, as this would strengthen the study’s theoretical contribution. - Provide a schematic or conceptual figure showing how identified themes map onto GT dimensions. This would help readers visualize how governance structures shape compliance. | Thank you for the important feedback. The discussion section has been revised to refine general statements to ensure all claims are supported by evidence and specific citations.  The integration of the institutional theory has been strengthened to show how it influences municipal compliance behaviour.  A conceptual model connecting determinants, strategies and compliance outcomes has been included.  Emphasis have been placed on explaining how municipal officials prefer the hierarchal approach, EMIs prefer command and control while EC advocate for collaboration or networked approach. |
| 7. Conclusion and Implications  - The conclusion is policy-relevant and forward-looking. - However, the recommendations are broad; prioritization would enhance their practical value. I would recommend specific actions tailored to different stakeholders (e.g., municipal managers, regulators, policymakers) and identify short- vs long-term priorities. | Thank you for the important feedback.  The conclusion has been refined and recommendations have been edited to be specific to municipal managers, regulators and policymakers. These have been categorised into short-term and long-term. |
| 8. Formatting, Clarity & Language  - Some editorial polishing is needed: - Overuse of passive voice, - Redundant wording in certain sections, - Occasional lapses in sentence clarity (e.g., lines 253–259). - The paper minor language and grammar editing, especially to improve sentence flow and eliminate verbosity. | Thank you for the important feedback. The manuscript has undergone language and style review to enhance clarity, readability and conciseness. Sentences written in passive voice have been revised to use active voice where appropriate. Redundant phrases have also been removed. |

| We appreciate the Editor’s positive assessment of our manuscript and the constructive feedback provided. We believe that the revisions have thoroughly addressed all identified shortcomings and have substantially strengthened the manuscript | |
| --- | --- |
| 1. Clarify research gap and originality compared to prior studies | Thank you for this critical feedback. We have revised the introduction to articulate the gap and contribution. We have rewritten the introduction to improve its coherence with the conclusion.  Prior studies focused on review of legislation with limited attention on how governance systems and institutional factors influence compliance. The rewritten introduction positions the study in relation to its contribution in the field of environmental governance. |
| 2. Strengthen theoretical framework linking governance theory to findings. | Thank you for the important feedback. We have expanded the theoretical framework to emphasise the connection between the GT and findings.  Emphasis have been placed on explaining how municipal officials prefer the hierarchal approach, EMIs prefer command and control while EC advocate for collaboration or networked approach. |
| 3. Provide justification for sample size of 27 participants. | Thank you for the important feedback. A paragraph has been added in the methodology section explaining that the sample of 27 participants was deemed adequate based on data saturation procedures proposed by Bekele and Ago (2022) and iterative analysis. |
| 4. Explain how municipalities were purposively selected. | Thank you for this critical feedback. We have added more details on how municipalities were selected. The criteria included their diversity, compliance history, environmental governance challenges, those with environmental units and those without. |
| 5. Include more detail on interview structure and questions. | Thank you for this critical feedback. A paragraph explaining how the interview structure was guided by a guide based on research objectives and the study's theoretical framework has been included. The details of how the study was piloted has also been included. The interview guide has also been included as annexure A. |
| 6. Discuss data reliability and potential researcher bias. | Thank you for the important feedback. A paragraph has been added in the methodology section describing the use procedures used to ensure trustworthiness. These included coding reliability, triangulation, member checking, audit trial, and transferability. During the analytical phase, authors also engaged in peer debriefing sessions and it has been included in the manuscript. |
| 7. Improve consistency in referencing recent South African studies. | Thank you for the important feedback. Although there is not many recent South African studies, some have been included such as Thakur and Nel 2024; Nzama 2023; Reddy 2018; Pasquini 2014 |
| 8. Expand on limitations and implications for generalizability. | We have added a section on limitations at the end of the manuscript to acknowledge the context bound nature of the findings and discussed its potential for transferability. |
| 9. Add a conceptual model connecting determinants and strategies. | Thank you for the important feedback. We have developed a conceptual model to visually illustrate how environmental compliance determinants and strategies influence compliance behaviour. Furthermore we have included some potential compliance outcomes that can be achieved. |
| 10. Quantitative analysis section lacks statistical clarity—revise. | Thank you for the important feedback. We have clarified the use of quantitative analysis on a qualitative study and we have edited the section to state that the analysis was descriptive and interpretive rather than inferential. |
| 11. Figures are under explained; improve captions and visual quality. | We have revised all figures for improved clarity and interpretive depth. The figure captions now provides more details relevant to the findings. |
| 12. Integrate discussion with international environmental governance literature. | We have integrated the discussion with international literature. The section now situate findings within the broader international frame. We looked at scholars in China and USA. The findings were compared particularly those in relation to political support. |
| 13. Ensure results and discussion are clearly differentiated. | We have separated the findings and discussion more clearly. With the quotations now reduced to allow more discussion and comparison with literature in the discussion. The revised sections now enhances logical flow and analytical clarity. |
| 14. Strengthen link between findings and policy recommendations. | Thank you for the important feedback. The conclusion and recommendations have now been revised to be specific to different stakeholders such as municipal managers, regulators and policymakers with short term and long term actions. |
| 15. Revise abstract for brevity and focus on main contributions. | We have revised the abstract and restructured it to sub-headings; Background, Methods, Results, Conclusion. It now highlights the purpose, methods and main findings. The redundant context have been removed. |
| 16. Clarify ethical considerations regarding participant anonymity. | More details have been given regarding the procedures followed to protect participants’ confidentiality, ensure informed consent and obtain institutional approval. |
| 17. Improve coherence between introduction and conclusion claims. | We have rewritten both the introduction and conclusion sections to ensure that the study’s aims and findings are conceptually aligned. The new sections provides a coherent narrative from the gap to theory and policy implications. |
| 18. Justify inclusion of quantitative content analysis in a qualitative design. | We have added justification for inclusion of quantified coding frequencies and co-occurrences to support qualitative interpretation and promote transparency. |
| 19. Provide citation support for claims about political influence. | We have strengthened the discussion regarding political influence and supported with various South African and international scholars. |
| 20. Revise for grammatical consistency and eliminate redundancy. | We have revised the manuscript to reduce passive voice, verbose phrasing and redundant expressions. Sentence flow has been revised to meet the journal requirements. |
